# Metamorphosis from Quantum Dots to Quantum Shells and Highly Efficient Quantum Shell Light–Emitting Diodes

**DOI:** 10.1002/advs.202505737

**Published:** 2025-06-23

**Authors:** Zhao Chen, Xiaohan Chen, Yuan Xiao, Shuming Ren, Yang Li

**Affiliations:** ^1^ Department of Basic Chemistry School of Pharmacy Zunyi Medical University Zunyi 563000 P. R. China; ^2^ School of Applied Physics and Materials Wuyi University Jiangmen 529020 P. R. China; ^3^ Poly Optoelectronics Tech. Ltd Jiangmen 529020 P. R. China; ^4^ Fujian Science & Technology Innovation Laboratory for Optoelectronic Information of China Fuzhou City 350108 P. R. China

**Keywords:** alloyed quantum shells, charge carrier dynamics, core/shell structures, efficient light–emitting diodes, high photoluminescence quantum yields

## Abstract

The bottom‐up design of chemical structure affords 0D nanocrystals (NCs) with tunable band structures and unexpected optical properties. Herein, an example of alloyed quantum shell (QS) is demonstrated by tailoring the chemical compositions in its core/shell structure. In the CdZnSe/ZnSeS/CdSeS/CdS (C/S_1_/S_2_/S_3_, in which C is the CdZnSe core and S represents the shells) structure, there is an intriguing metamorphosis from quantum dot (QD) to QS (that is, C and C/S_1_ belong to QDs, meanwhile C/S_1_/S_2_ and C/S_1_/S_2_/S_3_ are in the QS regime). Due to uniform morphology, perfect nanostructure, negligible defects, and unique energy level alignment, the C/S_1_/S_2_/S_3_ QS exhibits a high photoluminescence quantum yield of 90.9%, an ultra‐long fluorescence lifetime of 215.2 ns, and a slow radiative transition rate. It enables QS‐based light–emitting diodes (QS‐LEDs) with the state‐of‐the‐art performance, such as high external quantum efficiency (EQE of 22.16%) and excellent stability. Meanwhile, the investigation of charge carrier dynamics reveals the difference between the QD‐ and QS‐LEDs, showing that the charge carriers inside the QS‐LEDs need more time to recombine with each other. Based on these findings, this study believes that the emerging QSs can be attractive and efficient light–emitting materials used in lighting and displays.

## Graphical abstract

Herein, a novel quantum shell (QS) is achieved using an alloyed core/shell structure of CdZnSe/ZnSeS/CdSeS/CdS. It not only exhibits an intriguing metamorphosis from quantum dot to QS but also demonstrates outstanding photoluminescence and electroluminescence properties, such as high quantum yield, ultra‐long fluorescence lifetime, and high external quantum efficiency.

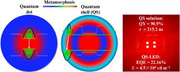

## 1 Introduction

Core/shell structured quantum dots (QDs) have received a significant interest due to their wide applications in biosensors, catalysis, photovoltaic devices, photodetectors, light–emitting diodes (LEDs), and other optoelectronic devices.^[^ ^1^, ^2^, ^3^, ^4^, ^5^, ^6^ ^]^ Their tunable energy band structures and optical properties (such as absorption and emission) achieved through the precise management of chemical composition enable a bottom‐up design for tailored functionality and performance.^[^ ^7^, ^8^ ^]^ It is well known that a typical CdSe/ZnS (where Cd, Se, Zn, and S represent cadmium, selenium, zinc, and sulfur, respectively) QD is characterized by a conventional type‐I energy level alignment, in which the energy gap gradually increases from the inner CdSe core to the outer ZnSe shell (Figure S1a, Supporting Information).^[^ ^9^ ^]^ The conventional type‐I QDs can easily achieve red, green, and blue emissions with near 100% photoluminescence quantum yields (PL QYs) and narrow emission spectra by tuning the size and chemical composition of the cores, and controlling the epitaxy of the shells.^[^ ^10^, ^11^, ^12^ ^]^ The QD‐LEDs based on the conventional type‐I QDs exhibit an impressive performance, such as high external quantum efficiencies (EQEs >20%), high brightness values (10^4^–10^6^ cd m^−2^), and long operation lifetimes.^[^ ^13^, ^14^, ^15^, ^16^, ^17^ ^]^ For example, Xu and coworkers reported an example of precisely designing the core/shell structured CdSe/ZnCdSe/ZnSe/ZnSeS/ZnS QD.^[^ ^13^ ^]^ In this case, the chemical composition of QD is gradually shifted from CdSe to ZnSe and ZnS through two alloy layers (ZnCdSe and ZnSeS) so as to release the lattice strain between CdSe wurtzite (WZ) and ZnSe (or ZnS) zinc blende (ZB). As a result, the CdSe/ZnCdSe/ZnSe/ZnSeS/ZnS QD exhibits a PL QY of over 95%. Meanwhile, a mixed‐crystallographic structure composed of WZ and ZB phases is observed in the core/shell structure of CdSe/ZnCdSe/ZnSe/ZnSeS/ZnS. The former is to promote dipole‐dipole interactions so as to achieve a special orientation and the latter lifts the electronic state degeneracy to enable a directional light emission from devices. It simultaneously achieves near 100% internal quantum efficiency and high photon out‐coupling efficiency, affording the state‐of‐the‐art QD‐LED based on the CdSe/ZnCdSe/ZnSe/ZnSeS/ZnS QD with a peak EQE of 35.6% and a *T*_95_ lifetime (corresponding to the time when the initial brightness value of 1000 cd m^−2^ decays to 950 cd m^−2^) of 4.5 years.^[^ ^13^ ^]^

On the contrary, ZnS/CdSe represents a reverse type‐I QD, in which the energy gap of the ZnS core is broader than that of the CdSe shell. As a result, the wave functions of charge carriers are confined within the CdSe shell, shifting the emission center into the CdSe shell (Figure S1b, Supporting Information).^[^ ^9^ ^]^ In this case, the emissive shells are exposed and susceptible to the surface defects and harmful environment, leading to the fact that the excited states of the reverse type‐I QDs are easily quenched and they are not in the list of the best candidates for electroluminescence (EL) devices.^[^ ^18^, ^19^, ^20^ ^]^ Fortunately, a strategy has emerged from an emerging material named quantum shells (QSs), in which further shell growth on the emissive shells of the reverse type‐I QDs protects the excited states from being quenched. This strategy can effectively address the challenge of the reverse type‐I QDs and improve their PL QYs.^[^ ^21^, ^22^ ^]^ Recently, several QS materials have been synthesized using the CdS/CdSe/CdS‐ and CdS/CdSe/CdS/ZnS‐based core/shell structures. The excitons generated from these QSs are confined inside a bulky CdSe shell geometry, leading to a spatial separation between multiple excitons and therefore ultra‐long biexciton lifetimes and high biexciton QYs under high‐intensity excitation. Therefore, the QS materials have a great potential to be used in lasers and X‐ray imaging.^[^ ^23^, ^24^ ^]^ To date, limited literature reports are known about the QS materials, and all of them are discrete core/shell structures. In addition, the QS materials have not yet been considered as emitters in QS‐LEDs.^[^ ^21^, ^22^, ^23^, ^24^, ^25^ ^]^

Herein, we demonstrate an example of alloyed QS with the core/shell structure of CdZnSe/ZnSeS/CdSeS/CdS. Intriguingly, there is an unusual metamorphosis from QD to QS after orderly growing the ZnSeS (S_1_), CdSeS (S_2_), and CdS (S_3_) shells on the surface of the CdZnSe core (C). It is noted that the C and CdZnSe/ZnSeS (C/S_1_) QDs feature the characteristic of the conventional type‐I energy level alignment so that their emissions are strongly dependent on the CdZnSe core.^[^ ^9^ ^]^ Meanwhile, the CdZnSe/ZnSeS/CdSeS (C/S_1_/S_2_) and CdZnSe/ZnSeS/CdSeS/CdS (C/S_1_/S_2_/S_3_) nanocrystals (NCs) exhibit an unconventional energy level alignment, in which the bandgap firstly increases then decreases from C to S_3_.^[^ ^16^, ^26^ ^]^ Importantly, the bandgap of S_2_ is smaller than those of C, S_1_, and S_3_, shifting the emission center from C to S_2_ and rendering that the C/S_1_/S_2_ and C/S_1_/S_2_/S_3_ NCs belong to QSs.^[^ ^21^ ^]^ The investigation of morphology, chemical composition, and optical property confirms the QD to QS metamorphosis. The CdZnSe, ZnSeS, and CdSeS alloys ensure a highly ordered epitaxy from the inner core to the outer shells, enabling the C/S_1_/S_2_/S_3_ QS to exhibit a perfect nanostructure and efficient surface passivation. It results in an excellent luminescence performance for the C/S_1_/S_2_/S_3_ QS, such as high PL QY of over 90% and ultra‐long fluorescence lifetime (τ) of over 210 ns. Meanwhile, the outermost S_3_ shell decreases the energy level offset between the highest occupied molecular orbital (HOMO) of hole transport material and the valence band (VB) energy level of QS, achieving balanced charge carriers inside the C/S_1_/S_2_/S_3_ QS emissive layer (EML).^[^ ^16^, ^26^ ^]^ As a result, the QS‐LED fabricated using the C/S_1_/S_2_/S_3_ QS achieves a peak EQE of 22.16% and a long operation lifetime under high initial luminance, representing a state‐of‐the‐art QS‐LED. Moreover, the differences between QD‐ and QS‐LEDs are revealed through the investigation of their charge carrier dynamics. The main difference is that the electrons and holes inside the EML of QS‐LED need much more time to recombine due to the bulky CdSeS shell geometry. Given the excellent PL and EL performance, we believe that the alloyed QSs will be novel and efficient light–emitting materials for lighting and displays.

## 2 Results and Discussion

### 2.1 Material Synthesis

The red‐emitting NCs were synthesized according to our previous reports.^[^ ^16^, ^26^ ^]^ Step A: In a 250 mL three‐necked flask, cadmium acetate dihydrate (Cd(OAc)_2_·2H_2_O, 0.6 mmol), zinc oxide (ZnO, 9 mmol), oleic acid (OA, 30 mL) and 1‐octadecene (ODE, 40 mL) were used as starting materials. The mixture was heated to 150 °C for 1h. Step B: It was heated to 300 °C and Se‐TBP (TBP is the abbreviation of tributylphosphine) was quickly injected into the reaction mixture under nitrogen (N_2_) to form the CdZnSe core (C). Step C: After 30 min, a 1 mL 1‐dodecanethiol (DDT) was added into the C solution and the reaction was kept at 300 °C for 30 min to grow the C/S_1_ NCs. Step D: A 5 mL 0.2 m Cd(OA)_2_ was quickly added into the C/S_1_ NCs. After 10 min, the C/S_1_ NCs were covered by the second shell to form the C/S_1_/S_2_ NCs. Step E: A 1 mL 4 m S‐TBP and 10 mL 0.2 m Cd(OA)_2_ were quickly added into the mixture and it was heated at 300 °C for 30 min to obtain the C/S_1_/S_2_/S_3_ NCs (the synthesis detail is shown in Supporting Information).

### 2.2 Morphology and Structure Studies of the C/S_1_/S_2_/S_3_ NCs

**Figure 1:**
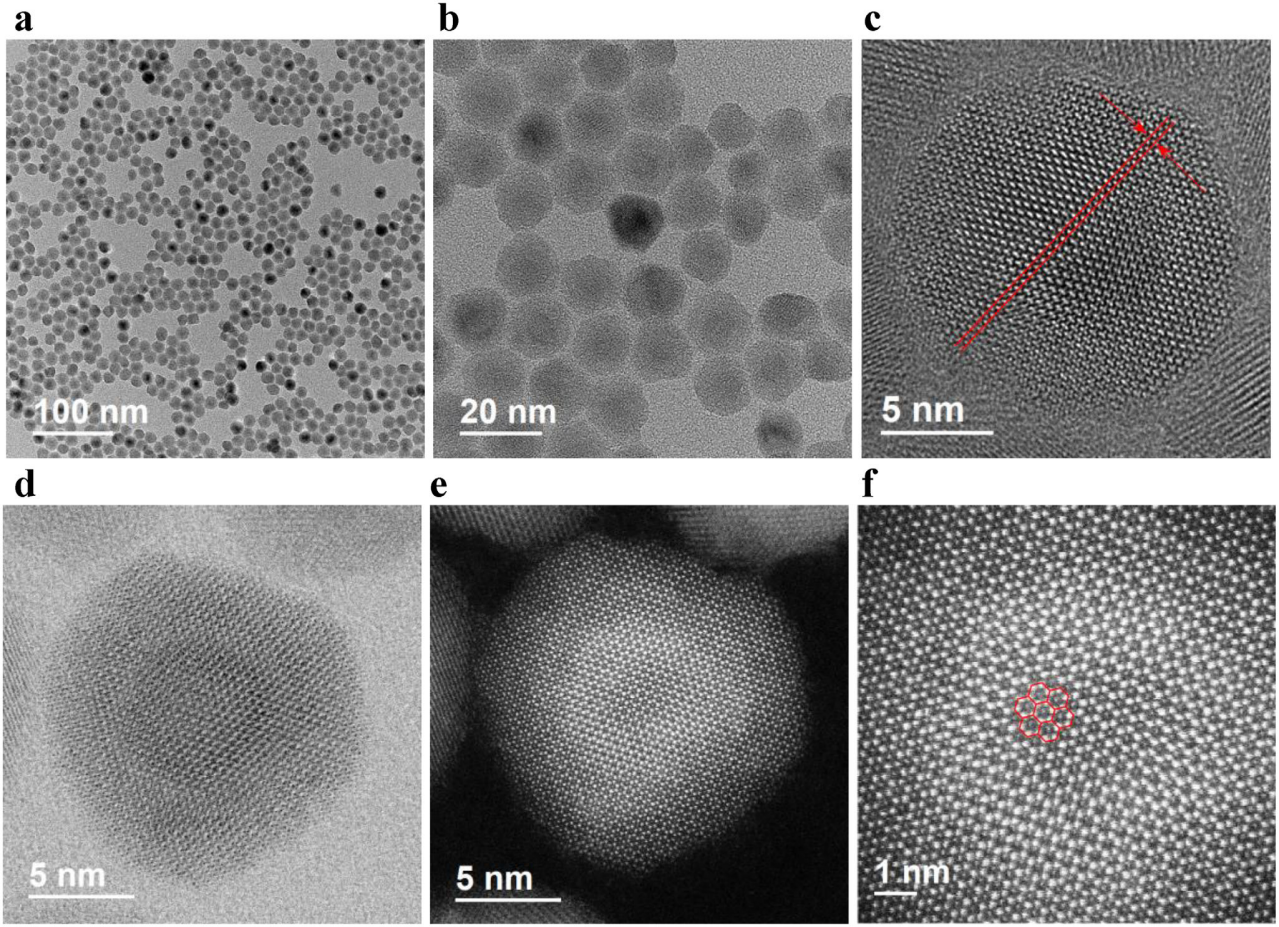
a–c) HR‐TEM images with different scale bars (the d space of ≈0.38 nm in Figure 1c), d,e) Bright‐ and dark‐field TEM images of a single NC, respectively. f) Atomically resolved HAADF‐STEM image.

The morphology of the C/S_1_/S_2_/S_3_ NCs was investigated using high‐resolution transmission electron microscope (HR‐TEM) and high‐angle annular dark‐field scanning TEM (HAADF‐STEM). As shown in **Figure** **1**a,b, these NCs exhibit a dot‐like morphology with a mean diameter of ≈15.28 nm (Figure S2, Supporting Information). Meanwhile, the diameter values of C, C/S_1,_ and C/S_1_/S_2_ are ≈8.98, 10.40, and 11.89 nm according to these HR‐TEM images shown in Figures S3–S5 (Supporting Information). It reveals that the thickness values of S_1_, S_2_, and S_3_ shells are ≈ 0.71, 0.75, and 1.70 nm, respectively. The size distribution of these NCs follows a Gaussian profile, exhibiting a slightly broad distribution range, such as ≈6–12 nm for the C NCs, ≈7–14 nm for the C/S_1_ NCs, ≈9–15 nm for the C/S_1_/S_2_ NCs, and ≈12–18 nm for the C/S_1_/S_2_/S_3_ NCs (Figures S2–S5, Supporting Information). In general, with an increasing number of statistical samples, the probability of observing both the smallest and largest particles gradually rises. Within the 300 samples, these instances are exceedingly rare, and their effect is insignificant. Under high magnification, the clear and regular crystal lattice fringes of C/S_1_/S_2_/S_3_ confirm its excellent crystallinity (Figure 1c).^[^ ^27^ ^]^ The lattices with a distance of ≈0.38 nm between each two crystal fringes correspond to the (100) plane of the hexagonal wurtzite (WZ) CdS (Figure 1c).^[^ ^28^ ^]^ The WZ crystal structure of C/S_1_/S_2_/S_3_ is further confirmed by the HAADF‐STEM and X‐ray diffraction (XRD) patterns (Figure 1d–f; Figure S6, Supporting Information).^[^ ^26^, ^28^ ^]^ To characterize the surface morphology of the C/S_1_/S_2_/S_3_ NCs at the atomic level, a single C/S_1_/S_2_/S_3_ dot in Figure 1d was analyzed using atomically resolved HAADF‐STEM. It is clearly observed that the hexagons formed by six bright points in Figure 1e,f are regularly and periodically extended from the center to the edge of the C/S_1_/S_2_/S_3_ dot, corresponding to no significant lattice mismatch during the epitaxy from the inner core to the outer shells.^[^ ^28^, ^29^, ^30^ ^]^ The uniform morphology and defect‐free surface structure (Figure 1a,f) confirm the superior crystal quality and nanostructural perfection of the C/S_1_/S_2_/S_3_ NCs.

The chemical compositions of C, C/S_1_, C/S_1_/S_2,_ and C/S_1_/S_2_/S_3_ were investigated using energy dispersive spectroscopy (EDS) and X‐ray photoelectron spectroscopy (XPS) (Figures S7–S9, Supporting Information). The elemental maps of Cd, Zn, Se, and S in the C and C/S_1_ NCs reveal that the Cd element distributes inside the inner core, and the Zn element locates at the surface. It corresponds to the CdSe inner core (size ≈ 6.60 nm) and the ZnSe (thickness ≈1.19 nm) (or ZnSeS, thickness ≈ 0.75 nm) outside shell (Figure S7a,b, Supporting Information). It is consistent with the previous reports, in which the chemical composition in the CdZnSe core exhibits a CdSe to ZnSe (or ZnSeS) transformation through a CdZnSe alloy transition layer.^[^ ^31^, ^32^, ^33^, ^34^ ^]^ According to the synthesis process and reaction kinetics, Cd elements with high reactivity preferentially react with Se elements to form CdSe. Upon complete consumption of Cd, the less reactive Zn subsequently reacts with Se, driving the epitaxial growth of ZnSe. The sequential reactions of Cd and Zn with Se ultimately yield a large CdZnSe core. Upon growth of the ZnSeS shell, the Cd element in the C and C/S_1_ NCs displays analogous spatial distribution, being effectively restricted to the inner core without substantial diffusion into the shells (Figure S7a,b, Supporting Information). The weight percentage (wt.%) of Zn element (30.1% and 33.8% for C and C/S_1_, respectively) is higher than that of Cd element (25.1% and 21.5% for C and C/S_1_, respectively) (Figure S8 and Table S1, Supporting Information), corresponding to the Zn‐rich C (CdZnSe) and C/S_1_ (CdZnSe/ZnSeS) NCs. After the epitaxy of S_2_ and S_3_, a significant increase in Cd wt.% (45.7% and 63.1% for C/S_1_/S_2_ and C/S_1_/S_2_/S_3_, respectively) is observed, accompanied by a decrease in Zn wt.% (16.2% and 8.8% for C/S_1_/S_2_ and C/S_1_/S_2_/S_3_, respectively) (Figure S8 and Table S1, Supporting Information). The quickly added Cd(OA)_2_ in the step D and E significantly increases the Cd wt.% values of C/S_1_/S_2_ and C/S_1_/S_2_/S_3_ due to the formation of the CdSeS (S_2_) and CdS (S_3_) shells. Different from our previous works, in which the slowly added Cd(OA)_2_ in the last step enables the growth of alloyed CdZnS shell and therefore enables a significant Zn distribution on the termination layer of materials, the element mapping signal of Zn on the surface of C/S_1_/S_2_/S_3_ is relatively weak according to the EDS results (Figure S7d, Supporting Information).^[^ ^16^, ^28^ ^]^ It is primarily due to the higher reactivity of Cd(OA)_2_ in comparison with Zn(OA)_2_ so that the Cd precursor in the step D and E preferentially reacts with Se or S precursors after a significant increase in Cd(OA)_2_ concentration, resulting in the formation of the Cd‐rich S_2_ and S_3_ shells.^[^ ^31^ ^]^

As illustrated in Figure S9 (Supporting Information), the XPS survey spectra exhibit the characteristic peaks corresponding to the constituent elements of the NCs, including Cd 3d, Zn 2p, Se 3d, Se 2p, and S 2p (Figure S9a, Supporting Information). In comparison with Se, the high electronegativity of S induces a shift in the Cd 3d peaks of the C/S_1_/S_2_ and C/S_1_/S_2_/S_3_ NCs toward higher binding energy (BE), attributed to the formation of Cd─S bonds in the CdSeS (S_2_) and CdS (S_3_) shells (Figure S9b, Supporting Information). A similar trend is observed in the Zn 2p spectra (Figure S9c, Supporting Information), which reflects the modified electronic structures of the metal element in the presence of S element.^[^ ^35^ ^]^ By comparing with the Cd 3d and Zn 2p spectra of these NCs, no significant BE peak shift is observed after S_2_ formation, corresponding to stable chemical environments of the metal elements, and suggesting that the formation of S_2_ and S_3_ does not facilitate significant exchange and diffusion between the Zn^2+^ and Cd^2+^ ions. These processes of the metal elements require overcoming the high bond dissociation energy (BDE) of their chalcogenide bonds, specifically, 127.6 ± 25.1 (Cd─Se), 208.5 ± 20.9 (Cd─S), 170.6 ± 25.9 (Zn─Se), and 224.8 ± 12.6 kJ mol^−1^ (Zn─S).^[^ ^36^ ^]^ Due to these substantial energy barriers, such processes typically occur under high‐temperature conditions, often facilitated by Cu^+^ ion catalysis.^[^ ^37^ ^]^ In this work, the element diffusion driven by ion concentration gradients at the interface of heterogeneous materials is found to be negligible even at high temperature, possibly leading to the formation of a thin transition alloy layer.^[^ ^38^ ^]^ As discussed above, the chemical composition of C/S_1_/S_2_/S_3_ structure gradually shifts from the Zn‐rich core to the Cd‐rich outer shells. It results in a possible core/shell structure of CdZnSe/ZnSeS/CdSeS/CdS for the C/S_1_/S_2_/S_3_ NCs by combining the synthesis process, reaction kinetics, and chemical composition. The alloyed core/shell structure facilitates the controlled NC growth, yielding uniform morphology and defect‐free surfaces (Figure 1).

### 2.3 Optical Properties of NCs

**Figure 2:**
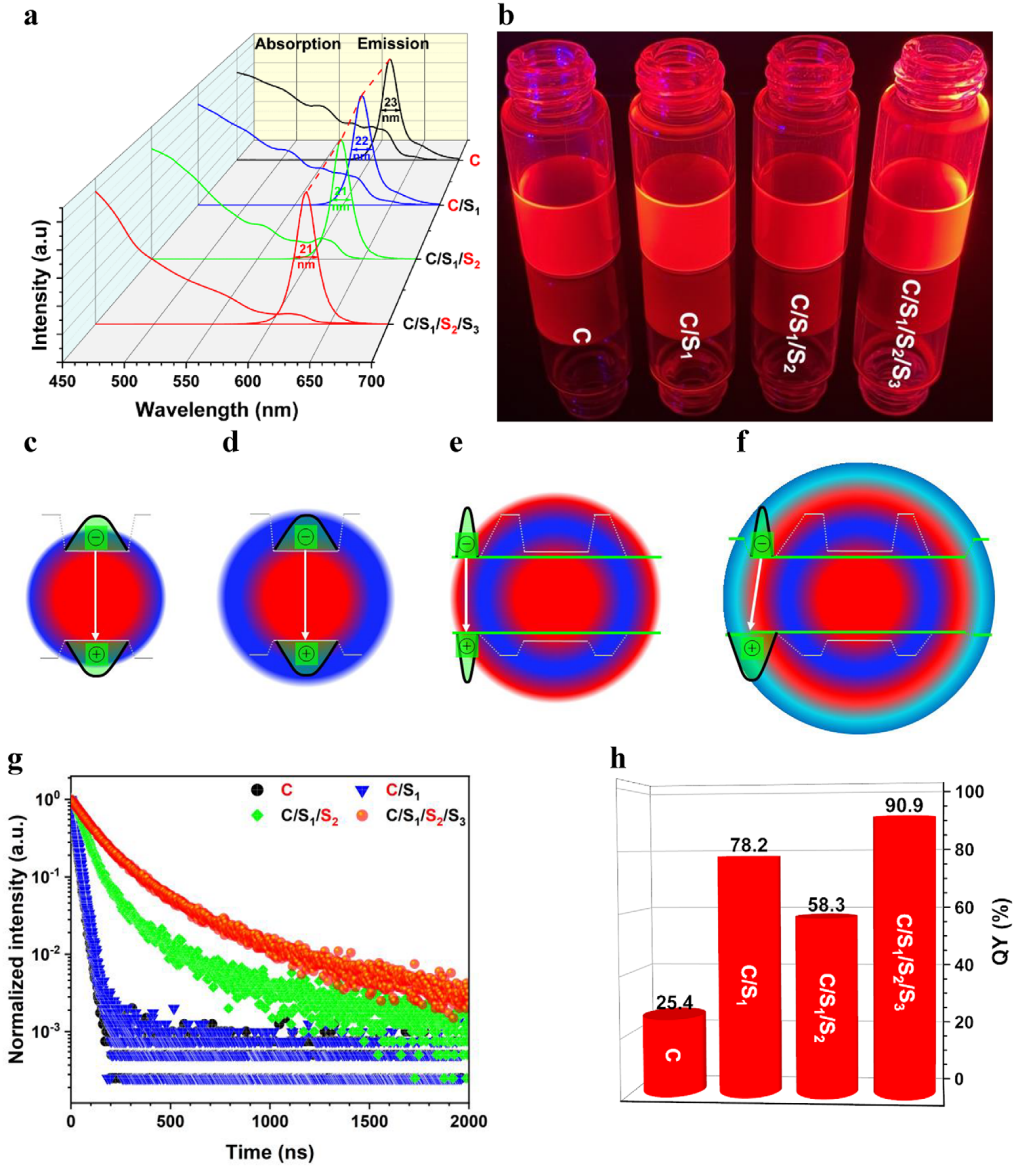
a) UV–vis absorption and PL spectra of the NC solutions. b) Images of NCs under a blue light (λ ≈ 460 nm). c–f) Schematic diagrams for the transformation from the conventional type‐I C and C/S_1_ QD (c,d) to the reverse type‐I C/S_1_/S_2_ QS (e) and the quasi‐type‐II C/S_1_/S_2_/S_3_ QS (f). g) PL decay curves of the NC solutions. h) Absolute PL QYs of the NC solutions.

To investigate the optical properties of these NCs, the absorption and emission spectra of the C, C/S_1_, C/S_1_/S_2_, and C/S_1_/S_2_/S_3_ NCs were measured. As shown in **Figure** **2**a, these NC solutions exhibit a significant self‐absorption with absorption tails up to their peak emission wavelengths due to the characteristic of band edge absorption and emission.^[^ ^39^, ^40^, ^41^ ^]^ It is noted that the peak emission wavelengths of C and C/S_1_ locate at 622 and 620 nm, which reveals that the growth of ZnSeS shell (S_1_) on the surface of CdZnSe core (C) has a slight influence on the emission of CdZnSe core. The slight Zn diffusion from S_1_ into the core may occur, resulting in a widened energy gap and therefore a blue‐shifted emission of C/S_1_.^[^ ^42^ ^]^ Intriguingly, the epitaxy of CdSeS (S_2_) and CdS (S_3_) significantly shifts the emissions of C/S_1_/S_2_ and C/S_1_/S_2_/S_3_ to a much longer wavelength region (emission peak at ≈630 nm) in comparison with C and C/S_1_ (Figure 2a,b; Table S2, Supporting Information). The full width at half maximum (FWHM) values of the C, C/S_1_, C/S_1_/S_2_, and C/S_1_/S_2_/S_3_ NCs are 23, 22, 21, and 21 nm, respectively (Figure 2a; Table S2, Supporting Information). Thanks to the symmetric Gaussian distribution, the influence of broad size variation in C/S_1_/S_2_/S_3_ on the emission spectrum is insignificant, resulting in well‐defined spectral symmetry and a small FWHM value (Figure 2a and Figure S10a, Supporting Information). The C/S_1_/S_2_/S_3_ NC demonstrates an outstanding PL stability, maintaining its emission intensity within the same order of magnitude after 15 days of UV irradiation (Figure S10b, Supporting Information). The optical properties of the NC films are shown in Figure S11 and Table S2 (Supporting Information). According to the XPS results, it is a challenge for Cd element to pass through the inner shells to significantly increase the Cd concentration and distribution area in the CdZnSe core.^[^ ^16^, ^28^, ^36^ ^]^ In this work, the observed redshift in PL spectra of C/S_1_/S_2_ and C/S_1_/S_2_/S_3_ may be attributed to migration of the emission center toward the Cd‐rich S_2_ shell region.

Essentially, this change is due to the chemical structure transformation from the Zn‐rich CdZnSe core to the Cd‐rich CdSeS shell. It results in a much narrower bandgap of Cd‐rich CdSeS shell (S_2_) in comparison with the Zn‐rich CdZnSe core so as to confine the charge carriers inside the light–emitting S_2_ shell.^[^ ^9^, ^21^, ^22^ ^]^ According to the results of ultraviolet photoelectron spectroscopy (UPS), the VB energy levels of C, C/S_1_, C/S_1_/S_2_, and C/S_1_/S_2_/S_3_ NCs are estimated at −6.65, −6.56, −5.97, and −6.04 eV, respectively, corresponding to the VBs of ZnSe, ZnSeS, CdSeS and CdS shells (Figure S12, Supporting Information).^[^ ^15^ ^]^ The CB energy levels are calculated based on the energy gaps of bulk materials, ≈−3.70 for ZnSe, −3.70 to −2.95 for ZnSeS, −4.00 for CdSeS, and −3.55 eV for CdS, respectively.^[^ ^9^, ^15^ ^]^ Based on these band structures, these NCs exhibit different energy level alignments and therefore diverse distributions of the electron and hole wave functions (Figure S13, Supporting Information). It is noted that the VB and CB energy levels of the CdZnSe core are entirely within those of the wide bandgap ZnSe and ZnSeS shells (Figure S13a,b, Supporting Information). The resulted quantum confinement effect confines the wave functions of charge carriers within the CdZnSe core (Figure 2c,d). Therefore, the C and C/S_1_ NCs exhibit a characteristic of conventional type‐I core/shell QDs.^[^ ^9^ ^]^ Intriguingly, the C/S_1_/S_2_ and C/S_1_/S_2_/S_3_ NCs exhibit a distinctive energy level alignment characterized by an initial increase followed by a decrease in bandgaps from the inner core to the outer shells (Figure S13c,d, Supporting Information).^[^ ^43^ ^]^ The CB and VB energy level of the S_2_ (CdSeS) shell are entirely encompassed by those of C (CdZnSe) and S_1_ (ZnSeS), corresponding to a reverse type‐I energy alignment of the C/S_1_/S_2_ structure (Figure 2e).^[^ ^9^, ^43^, ^44^ ^]^ It is noted that the S_2_ shell possesses a much deeper CB and a well‐matched VB energy level compared to the S_3_ (CdS) shell (Figure S13d, Supporting Information). According to these discussions, the wave function of electron in the C/S_1_/S_2_/S_3_ structure is entirely confined within the S_2_ shell due to its deeper CB energy level in comparison with the core and the shells. Meanwhile, the wave function of hole may diffuse into the entire S_2_ and S_3_ shells due to their aligned VB energy levels, corresponding to a quasi‐type‐II energy level alignment (Figure 2f).^[^ ^45^ ^]^ From the inner C to the outermost S_3_ shell, a remarkable transformation of energy level alignment from the conventional type‐I to reverse type‐I and quasi‐type‐II occurs (Figure 2c–f). Based on their chemical composition, energy level alignment, and PL emission features, the C and C/S_1_ NCs can be classified as QDs, whereas the C/S_1_/S_2_ and C/S_1_/S_2_/S_3_ belong to QSs. Fortunately, the delocalization of hole within the S_2_ and S_3_ shells has minimal influence on the emission wavelength and FWHM of the C/S_1_/S_2_/S_3_ structure, as the bandgaps from the CB energy level of the S_2_ shell to the VB energy levels of the S_2_ and S_3_ shells are nearly identical (Figure S13d, Supporting Information).

The excited states of QSs exhibit substantially long average lifetimes (τ_av_) of 137.6 and 215.2 ns for the C/S_1_/S_2_ and C/S_1_/S_2_/S_3_ QSs, respectively, significantly longer than those of the C (τ_av_ = 22.1 ns) and C/S_1_ (τ_av_ = 26.2 ns) QDs (Figure 2g). This dramatic difference originates from distinct carrier confinement effects: In the C and C/S_1_ QDs, strong quantum confinement within the spherical core forces complete spatial overlap between electron and hole wavefunctions, accelerating electron–hole recombination and consequently shorter τ values.^[^ ^46^ ^]^ In contrast, the bulky geometry of QS system promotes spatial separation of exciton. The quantum confinement in the S_2_ shell reduces the possibility of electron–hole encounters.^[^ ^47^ ^]^ Meanwhile, additional hole delocalization into the S_3_ shell further enhances the separation effect in the C/S_1_/S_2_/S_3_ QS.^[^ ^48^ ^]^ These combined factors ultimately yield the prolonged lifetime of 215.2 ns for the C/S_1_/S_2_/S_3_ QS. The absolute QYs of the C, C/S_1_, C/S_1_/S_2_, and C/S_1_/S_2_/S_3_ NCs also exhibit an unexpected trend. In theory, the QYs of the core/shell structures generally increase due to the efficient shell passivation. Intriguingly, the QY of the C/S_1_/S_2_ QS (QY = 58.3%) is lower than that of the C/S_1_ QD (QY = 78.2%) and the QY of the C/S_1_/S_2_/S_3_ QS increases to 90.9% after the growth of the outermost S_3_ shell (Figure 2h). The decreased QY of the C/S_1_/S_2_ QS is primary due to the shift of emission center from the inner core to the outer shell, exposing the emissive S_2_ shell to the surface defects and harmful environment in the absence of efficient shell passivation. Consequently, the excited state of the C/S_1_/S_2_ QS is easily quenched, resulting in a relatively low PL QY.^[^ ^9^ ^]^ Fortunately, the outermost S_3_ shell can efficiently passivate the emissive S_2_ shell. On the one hand, the alloyed core/shell structure of the CdZnSe/ZnSeS/CdSeS/CdS QS facilitates the ordered epitaxial growth of these heterogeneous materials (from CdZnSe to CdS), effectively minimizing interfacial defects. On the other hand, the large‐size CdZnSe core expands the overall dimensions of the C/S_1_/S_2_/S_3_ QS (size = 15.28 nm), synergistically passivating surface defects in conjunction with the perfect WZ CdS surface structure (Figure 1e,f). Therefore, it is reasonable that the C/S_1_/S_2_/S_3_ QS achieves a high QY of over 90%. In addition, the radiative transition rate (k_r_) constant of the C/S_1_/S_2_/S_3_ QS calculated using the Equations S4 and S5 (Supporting Information) is ≈4.2 × 10^6^ s^−1^ (Table S2, Supporting Information), one order of magnitude smaller than those of core/shell structured QDs.^[^ ^26^ ^]^ It corresponds to a slow radiation process of the C/S_1_/S_2_/S_3_ QS. Therefore, the C/S_1_/S_2_/S_3_ QS possesses unexpectedly optical properties, such as red‐shifted emission, ultra‐long fluorescence lifetime, and slow radiation decay. Given the substantial impact of core size and emitting shell thickness on the emission properties of these QS materials, ongoing research aims to uncover their precise relationship.

### 2.4 QS‐Based Devices

#### 2.4.1 Device Structure and Performance of QS‐LED

**Figure 3:**
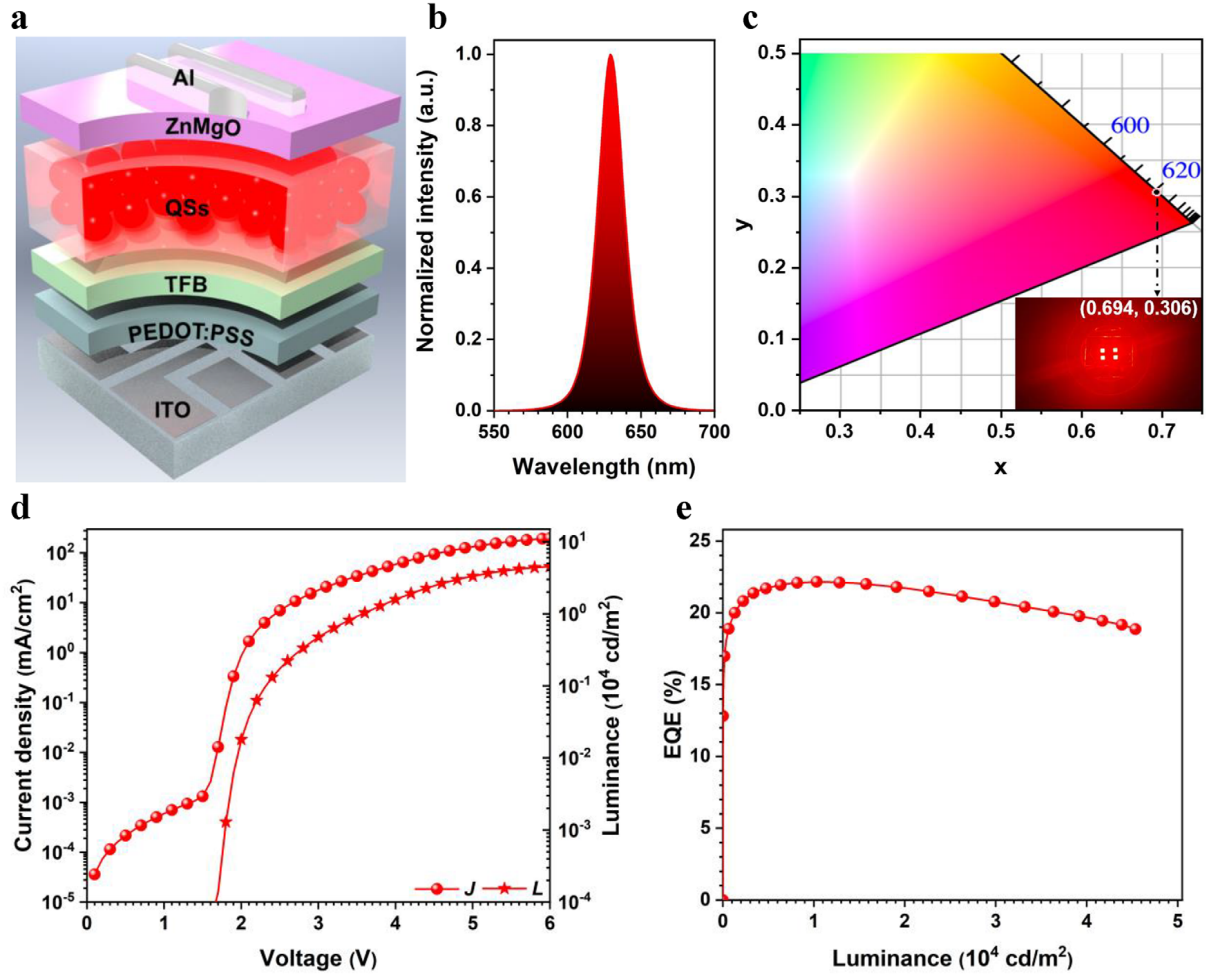
a) Device structure of QS‐LED. b) EL spectra of red‐emitting QS‐LED. c) CIE coordinates (The inset image exhibits the red emission from a working device). d) *J*–*V*–*L* curve. e) EQE−*L* curve.

The QS‐LED was fabricated according to the device architecture of ITO/PEDOT:PSS (30 nm)/TFB (40 nm)/QS EML (20 nm)/ZnMgO (40 nm)/Al (100 nm) (**Figure** **3**a). In the device structure, ITO (indium tin oxide) and Al (aluminum) are used as anode and cathode, respectively. PEDOT:PSS (poly(3,4‐ethylenedioxythiophene):poly(styrenesulfonate)) is functionalized as hole injection layer (HIL). TFB (poly(9,9‐dioctylfluorene‐*co*‐*N*‐(4‐(*sec*‐butyl)phenyl)diphenyl‐amine) and ZnMgO (magnesium doped zinc oxide nanoparticles) play the role of hole and electron transport layer (HTL and ETL), respectively.^[^ ^49^, ^50^, ^51^ ^]^ The detail of device fabrication is shown in Supporting Information. The red‐emitting QS‐LED exhibits a peak emission wavelength of ≈630 nm with a FWHM of 22–24 nm (Figure 3b; Figure S14b, Supporting Information), corresponding to the Commission Internationale de l'Eclairage (CIE) chromaticity coordinates of ≈(0.694, 0.306) (Figure 3c). When the applied voltage is increase to 6 V, no significant shift of peak emission wavelength in the EL spectrum is observed, corresponding to a stable red emission (Figure S14a,b, Supporting Information). The EL spectrum of the QS‐LED closely matches that of the C/S_1_/S_2_/S_3_ QS measured in film (Figure S14c, Supporting Information), confirming that the red emission is originated from the QS EML.

Due to the Cd‐rich outside shells, the VB energy level of QS is shifted to ≈−6.0 eV, which aligns well with the HOMO of TFB (HOMO_TFB_ = −5.33 eV). Therefore, the EL device can be lighted with a low applied voltage of ≈1.7 V, corresponding to a turn‐on voltage (*V*_on_) of 1.7 V for the QS‐LED (Figure 3d). The luminance (*L*) value can reach 4.5 × 10^4^ cd m^−2^ when the applied voltage is increased to 6 V. The peak current, power, and external quantum efficiencies (CE, PE, and EQE) are recorded at 26.98 cd A^−1^, 32.96 lm W^−1^ and 22.16%, respectively (Figure 3e; Figure S15c and Table S3, Supporting Information). It is noted that the peak EQE is achieved at the luminance of 1.03 × 10^4^ cd m^−2^ and it is over 20% when the luminance is increased to 3.64 × 10^4^ cd m^−2^, revealing that the QS‐LED in this work exhibits a restricted EQE roll‐off (Figure 3e). Meanwhile, the device based on the CdZnSe/ZnSeS/CdZnS QD was fabricated to compare the performance of the QS‐ and QD‐LEDs. The CdZnSe/ZnSeS/CdZnS QD was reported in our previous work.^[^ ^26^ ^]^ It was selected due to its size of ≈15.2 nm, PL QY of 96.7%, and VB energy level of −6.02 eV, which match well with those of the CdZnSe/ZnSeS/CdSeS/CdS QS (size = 15.3 nm, PL QY = 90.9% and VB = −6.04 eV). As shown in Figure S15 (Supporting Information), the QD‐LED exhibits a luminance of over 1.0 × 10^5^ cd m^−2^ at 6 V, which is twice that of the QS‐LED (*L* = 4.5 × 10^4^ cd m^−2^). It requires much more electrons to recombine with the injected holes inside the QD‐LED to achieve such high luminance (Figure S15b, Supporting Information). As a result, the peak EQEs of these devices exhibit no significant distinction, 22.16% for the QS‐LED and 21.78% for the QD‐LED (Figure S15d, Supporting Information). A statistical evaluation of the EQE across 60 devices reveals that every device surpasses 20%, with a mean EQE of 21.71%, indicating exceptional device‐to‐device performance reproducibility (Figure S16, Supporting Information). In addition, when the initial luminance (*L*_0_ > 3.0 × 10^4^ cd m^−2^) decays to half of its value, the *T*_50_ lifetimes of the QD‐ and QS‐LEDs are ≈81.2 and 37.3 h, respectively (Figure S17 and Table S3, Supporting Information). Therefore, when comparing the performance of these light–emitting devices, their efficiencies are nearly identical, and the QD‐LED is superior to the QS‐LED in terms of luminance and operational lifetime. Nevertheless, this work not only represents the first QS‐LED but also establishes the state‐of‐the‐art performance in this emerging field, demonstrating that the QS materials have the potential to be used in high‐efficiency and stable light–emitting devices.

#### 2.4.2 Charge Carrier Dynamics of Light–Emitting Devices

To further distinguish the QD‐ and QS‐LEDs, the charge carrier dynamics inside these devices were investigated. On the one hand, the electron‐ and hole‐only devices (EODs and HODs) based on the CdZnSe/ZnSeS/CdZnS QD and the CdZnSe/ZnSeS/CdSeS/CdS QS were fabricated, and their *J*−*V* curves were tested (Figure S18, Supporting Information). It is noted that the current densities of EODs and HODs based on the same material are well‐matched at the same applied voltages, confirming that the charge carriers in the QD‐ and QS‐LEDs are balanced (Figure S18e, Supporting Information).^[^ ^16^, ^26^, ^48^ ^]^ It corresponds to the values of change carrier balance factor (*γ*) in these LEDs are nearby 100%. As is well established, the EQEs of light–emitting devices can be calculated according to the equation of EQE = *γ* × *η*_int_ × *η*_out_, where *η*_int_ and *η*_out_ represent the internal quantum efficiency and light out‐coupling efficiency, respectively. In general, the PL QYs of the emitters approximately equals to the *η*_int_ values, while *η*_out_ approaches ≈30% in standard planar optical configurations.^[^ ^52^ ^]^ As a result, the QD‐ and QS‐LEDs own high EQEs of over 20% due to the high PL QYs (*η*_int_ ≈ 90%) of the emitters and the balanced charge carriers (*γ* ≈ 100%) inside the EMLs. It is found that the EOD and HOD based on the CdZnSe/ZnSeS/CdZnS QD exhibit higher current densities than those of the devices made by the CdZnSe/ZnSeS/CdSeS/CdS QS (Figure S18e, Supporting Information). It indicates that more charge carriers are injected and transported into the CdZnSe/ZnSeS/CdZnS QD EML, leading to higher luminance of the QD‐LED than that of the QS‐LED. On the other hand, the electrochemical impedance spectroscopy (EIS) Nyquist plots of the QD‐ and QS‐LEDs were obtained (Figure S19, Supporting Information). An equivalent circuit containing *R*_s_, *R*_tr_ and *R*_rec_ was used to fit these Nyquist plots (the inset in Figure S19c, Supporting Information), where these resistances correspond to the overall external resistance (*R*_s_, originating from electrodes, connecting wires, and interfaces between the electrodes and functional layers), charge transfer based resistance (*R*_tr_), and recombination resistance (*R*_rec_), respectively (Figure S19a,b, Supporting Information).^[^ ^26^, ^51^, ^53^ ^]^ The QD‐LED exhibits much smaller *R*_tr_ (42 Ω m^−2^) and *R*_rec_ (74119 Ω m^−2^) values than the QS‐LED (*R*_tr_ = 146 Ω m^−2^ and *R*_rec_ = 128116 Ω m^−2^) at 1.7 V (Figure S19c and Table S4, Supporting Information). It confirms that the transport and recombination of electrons and holes inside the QD‐LED are more efficient. Meanwhile, the larger R values increase the Joule heat under high current densities, accelerating device aging at high luminance values, and resulting in EQE roll‐off and short operation lifetime of the QS‐LED (Figures S15d and S17b, Supporting Information).

**Figure 4:**
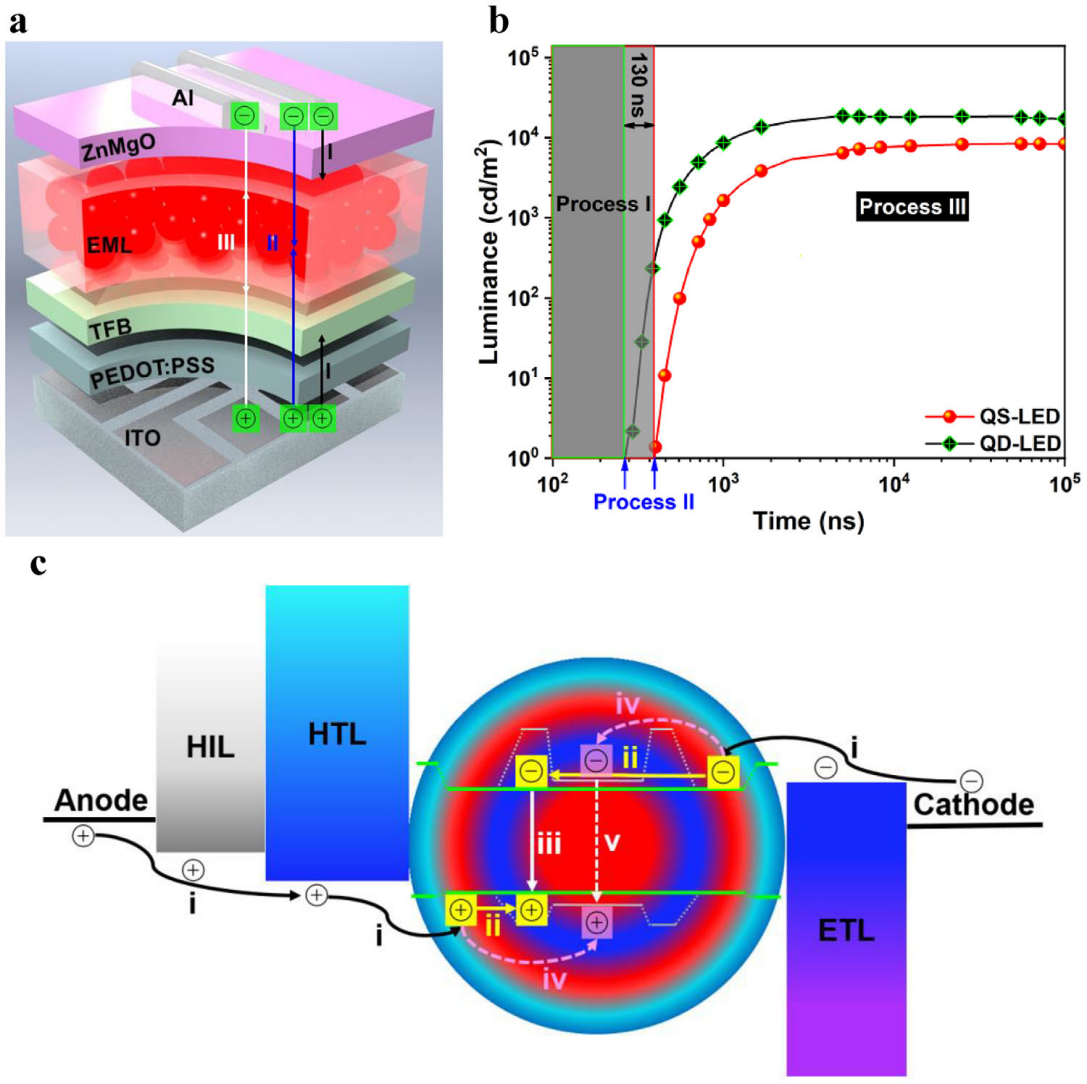
a) Charge carriers in the light–emitting devices under different frequencies (Process I: Under a high‐frequency electrical excitation, the electrons and holes do not have enough time to reach the QS EML, resulting in no photon emission from the LEDs. Process II: Under a critical frequency, the charge carriers are injected into the EML, generating photons and enabling the detection of the emission spectra from the LEDs. Process III: A large number of charge carriers are injected into the EML to dramatically increase the number of photons when the frequency is significantly lower than the critical one. In this case, the luminance of the LEDs gradually reaches its maximum.). b) *L*−t curves of LEDs measured at 4 V. c) Schematic diagram of the charge carrier dynamics in the QS‐LED.

Meanwhile, the time between the injection and recombination of charge carriers inside the light–emitting devices was measured according to the mode shown in **Figure** **4**a.^[^ ^54^ ^]^ If the light–emitting devices are driven by an electric field with a square wave signal (Figure S20, Supporting Information), there are three possible scenarios for charge carriers in Figure 4a. In process II, the critical frequency is defined here as the frequency corresponding to the device brightness of 1 cd m^−2^. The corresponding time (t) for the injection, transport, recombination of charge carriers, and light emission is equal to half of the critical electricity signal period (T) (Figure S20b, Supporting Information). As shown in Figure 4b; Figure S21 (Supporting Information), the t values decrease as the applied voltage increases from 3 to 5 V. For example, the t values of the QS‐LED are ≈658, 394, and 325 ns at 3, 4, and 5 V, respectively, and those of the QD‐LED are ≈486, 264, and 224 ns at 3, 4, and 5 V, respectively (Figure 4b; Figure S21, Supporting Information). On the one hand, the shorter t values at higher applied voltage are reasonable since the processes containing the injection and transport of charge carriers can be accelerated by increasing the applied voltage.^[^ ^55^ ^]^ On the other hand, it is intriguing to find that t values for the QS‐LED are ≈100 to 200 ns longer than those for the QD‐LED, which is close to the τ value of the CdZnSe/ZnSeS/CdSeS/CdS QS film (τ = 135.2 ns). Due to the same functional layers (such as HIL, HTL, ETL, and electrodes) used in these device architectures, the different light–emitting materials inside the EMLs have a great influence on the t values. There are two distinctions between the QD‐ and QS‐EMLs. First, the shortest distance that the electrons and holes need to transfer from the surface of the CdZnSe/ZnSeS/CdSeS/CdS QS to the recombination site is ≈21.23 nm (Figure S22b, Supporting Information), which is slightly longer than that of the CdZnSe/ZnSeS/CdZnS QD (≈15.20 nm, Figure S23b, Supporting Information). However, since the QD‐ and QS‐LEDs can be turned on at the same *V*_on_ (1.7 V), we believe that this point contributes less to the distinctions. Second, the injected electrons and holes inside the QS EML are hard to find each other so that they need more time to recombine in the bulky geometry of the CdSeS shell (Figure S22c, Supporting Information). The additional time of 100–200 ns is attributed to the time needed for the charge carriers to locate each other within the CdSeS shell geometry. It is consistent with the longer τ (135.2 ns) and slower k_r_ rate (6.0 × 10^6^ s^−1^) of the CdZnSe/ZnSeS/CdSeS/CdS QS film (Table S2, Supporting Information), as well as the larger R_rec_ (128116 Ω m^−2^ at 1.7 V) of the QS‐LED compared to the CdZnSe/ZnSeS/CdZnS QD film (τ = 15.9 ns and k_r_ = 3.8 × 10^7^ s^−1^) and the QD‐LED (*R*_rec_ = 74119 Ω m^−2^ at 1.7 V).

Based on these discussions, the proposed charge carrier dynamics of the QS‐LED involve the injection and transport of charge carriers from the electrodes to EMLs (process i in Figure 4c). Intriguingly, these injected charge carriers fail to recombine immediately to produce the photons. An additional transport step (process ii) of charge carriers inside the CdSeS shell is required since they are confined in the bulky shell geometry. The charge carriers prefer to meet and recombine, subsequently converting to light inside the CdSeS shell (process iii) rather than being further injected into the CdZnSe core through process iv as there is a significant energy offset between the CdSeS shell and the ZnSeS shell (Figure 4c). In contrast to the typical dynamics in the QD‐LED (Figure S23a, Supporting Information), the exciton generation inside the CdZnSe core (process v in Figure 4c) may not occur in the QS‐LED.^[^ ^56^, ^57^ ^]^ Based on the above mechanism, the emission of the QS‐LED originates from the CdSeS shell rather than the CdZnSe core.

## 3 Conclusion

Herein, a novel QS with an alloyed core/shell structure of CdZnSe/ZnSeS/CdSeS/CdS (C/S_1_/S_2_/S_3_) is achieved. According to the EDS results, there is a transformation of chemical composition from the Zn‐rich C and C/S_1_ to the Cd‐rich C/S_1_/S_2_ and C/S_1_/S_2_/S_3_ after orderly covering the CdZnSe core with the S_1_, S_2_ and S_3_ shells. The investigation of the energy band structure reveals that the C and C/S_1_ NCs exhibit characteristics of the conventional type‐I QDs. In contrast, the C/S_1_/S_2_ and C/S_1_/S_2_/S_3_ NCs are classified as the reverse type‐I and quasi‐type‐II QSs, respectively. The QD‐to‐QS metamorphosis well responses to the differences in the optical properties of these NCs, such as red‐shifted emission wavelengths, longer lifetimes of excited states, and slower radiative transition decay rates of C/S_1_/S_2_ and C/S_1_/S_2_/S_3_. Due to uniform morphology, perfect surface nanostructure, less lattice mismatch, and efficient shell passivation, the C/S_1_/S_2_/S_3_ QS exhibits excellent optical properties, such as pure red emission (λ = 630 nm), narrow FWHM of 21 nm, ultra‐long τ of 215.2 ns, and high QY of 90.9%. Meanwhile, the electroluminescent QS‐LED exhibits a high EQE of 22.16% and a long *T*_50_ lifetime of 37.3 h (measured at an initial brightness of ≈3.3 × 10^4^ cd cm^−2^), corresponding to the most efficient QS‐LED. The difference between the devices fabricated using the CdZnSe/ZnSeS/CdSeS/CdS QS and the CdZnSe/ZnSeS/CdZnS QD is investigated. On the one hand, it is noted that the luminance and operation lifetime of the QD‐LED surpass those of the QS‐LED, and the EQEs of these devices are nearly identical. On the other hand, the charge carriers in the QS EML need additional time (100−200 ns) to locate each other and then generate photons due to the bulky geometry of the CdSeS shell. This behavior contrasts with the typical dynamics of charge carriers in the QD EML, where the photons are immediately generated upon the injection of charge carriers into the QD EML. Based on these results, we believe that the alloyed QSs represent emerging, attractive, and efficient light–emitting materials used in lighting and displays.

## Conflict of Interest

The authors declare no conflict of interest.

## Supporting information



Supporting Information

## Data Availability

The data that support the findings of this study are available from the corresponding author upon reasonable request.
