# Supplementary material for: Metamorphosis from Quantum Dots to Quantum Shells and Highly Efficient Quantum Shell Light–Emitting Diodes
Source: Adv Sci (Weinh). 2025 Jun 23;12(35):e05737. doi: 10.1002/advs.202505737 (PMC12462930; doi:10.1002/advs.202505737)
Supplement: Supplementary file 1 — Supporting Information [file ADVS-12-e05737-s001.docx]

Supporting Information

Metamorphosis from quantum dots to quantum shells and highly efficient quantum shell light-emitting diodes

*Zhao Chen*, Xiaohan Chen, Yuan Xiao, Shuming Ren, Yang Li*

Dr. Z. Chen and Dr. Y. Xiao

Department of Basic Chemistry, School of Pharmacy, Zunyi Medical University, Zunyi, 563000, P. R. China

E-mail: chenzhao@zmu.edu.cn

Dr. Z. Chen and X. Chen

School of Applied Physics and Materials, Wuyi University, Jiangmen, 529020, P. R. China

Dr. Y. Li

Fujian Science & Technology Innovation Laboratory for Optoelectronic Information of China, Fuzhou City, 350108, P. R. China

Dr. Z. Chen, S. Ren and Dr. Y. Li

Poly Optoelectronics Tech. Ltd, Jiangmen, 529020, P. R. China

1. **Experimental Section**

**Materials**

Cadmium acetate dihydrate (Cd(OAc)_2_·2H_2_O), zinc oxide (ZnO), selenium (Se), sulfur (S), oleic acid (OA), octadecene (ODE), tributyl phosphine (TBP), 1-dodecanethiol (DDT), octane, n-hexane, ethyl acetate, ethyl alcohol and chlorobenzene (CB) were purchased from Sigma-Aldrich. The 0.2M Cd(OA)_2_, 2M Se-TBP and 4M S-TBP precursors were prepared according to our previous works.^[1−4]^ The functional materials used in light-emtting devices such as poly(3,4-ethylenedioxythiophene):poly(styrene-sulfonate) (PEDOT:PSS) hole injection material, poly(9,9-dioctylfluorene-*co*-*N*-(4-(*sec*-butyl)phenyl)diphenylamine) (TFB, M_w_ = ~80 kDA) hole transport material and magnesium doped zinc oxide (ZnMgO) electron transport material were purchased from Luminescence Technology Corp., American Dye Source and Guangdong Poly OptoElectronics Co., Ltd, respectively. Indium tin oxide (ITO, 30 Ω sq^−1^) substrates and aluminum were purchased from Wuhu Jinghui Electronic Technology Co., Ltd. and Beijing Dream Material Technology Co., Ltd.

**Material synthesis**

ZnO (9 mmol, 733 mg) and Cd(OAc)_2_·2H_2_O (0.6 mmol, 160 mg), 30 mL OA and 40 mL ODE were placed into a 250 mL container. After dehydration and degassing at 150 ^o^C for 1 hour, the temperature was quickly increased to 300 ^o^C and a 1 mL 2M Se-TBP was immediately injected into the mixture under N_2_. After 30 minutes, the CdZnSe core (C) solution was obtained. Then, a 1 mL DDT was injected into the mixture and the ZnSeS (S_1_) alloy shell was formed on the C surface after 30 minutes. Cd(OA)_2_ (0.2M, 5 mL) was quickly added into the mixture and the reaction was kept at 300 ^o^C for 10 min. After the formation of C/S_1_/S_2_, Cd(OA)_2_ (0.2M, 10 mL) and S-TBP (4M, 1 mL) precursors were quickly injected into the C/S_1_/S_2_ solution. After 30 minutes, the reaction was quenched by cooling down the reaction temperature and the C/S_1_S_2_/S_3_ nanocrystals (NCs) were obtained. To purify these NCs, a mixture of n-hexane and ethyl acetate was added into the NC solutions when the reaction temperature decreased to room temperature (RT). Then, the precipitate was collected by centrifugation at the speed of 6000 rpm and it was redispersed into n-hexane and reprecipitated with ethyl acetate by three times. These precipitates were redispersed into octane to prepare the NC solutions (30 mg mL^−1^).^[1−4]^

**Device fabrication**

The ITO substrates (30 Ω sq^−1^) were washed in an ultrasonic cleaner by orderly using detergent and deionized water by three times. To fabricate the PEDOT:PSS hole injection layer (HIL), the surface of ITO substrate was firstly treated with UV-O_3_ for 45 min. Then, a 30 nm HIL was fabricated on the ITO surface by spin-coating the PEDOT:PSS aqueous solution with a speed of 3000 rpm, and the PEDOT:PSS film was annealed under 150 °C for 15 min in air. In a N_2_ filled glove box, a 40 nm TFB hole transport layer (HTL) was vertically stacked on the surface of PEDOT:PSS by spin-coating a TFB solution (8 mg mL^−1^ in CB) with the speed of 3000 rpm. The film was placed on a hot plate and baked at 120 °C for 10 min. A CdZnSe/ZnSeS/CdSeS/CdS quantum shell (QS) or CdZnSe/ZnSeS/CdZnS quantum dot (QD) emissive layer (EML, about 20 nm) was fabricated by spin-coating the QS or QD solution (15 mg mL^−1^ in octane) on the surface of TFB HTL with the speed of 3000 rpm, and the EML film was baked at 60 °C for 5 min. By spin-coating the ZnMgO solution (20 mg mL^−1^ in ethanol alcohol) with the speed of 3000 rpm, a 40 nm ZnMgO electron transport layer (ETL) was fabricated on the EML surface. The ZnMgO film was annealed at 60 °C for 10 min. Under a pressure of smaller than 2×10^−6^ torr, 100 nm Al cathode was fabricated through thermal evaporation. The devices with an active area of 4 mm^2^ were encapsulated using a glass cover and UV glue before transferring them into air.^[1−4]^

**Characterization and** **instrumentation**

The absoprtion and emission spectra of NC solutions and films were recorded on an UV-visible absorption spectrometer (Hitachi U-3900H) and a steady-/transient-state fluorescence spectrometer (Edinburgh Instruments Spectrometer FSL980). For UV-visible absorption test, the scanning wavelength is in the region of 200−800 nm with a step of 1 nm. The light sources containing a xenon lamp and light-emitting diode were used to obtain the photoluminescence (PL) spectra and the excitation wavelength was 375 nm. The emission spectra were scanned from 395 to 780 nm with the step of 1 nm. The decay lifetimes (τ_i_) and the corresponding constants (A_i_) could be obtained by fitting the transient PL decay curves using the equation 1:

I(t) = A_1_e^−t/τ1^ + A_2_e^−t/τ2^ + A_3_e^−t/τ3^ + ··· 1

The average lifetimes (τ_av_) could be calculated according to the equation 2:

τ_av_ = $\frac{\sum_{1}^{i} A_{i}\tau_{i}^{2}}{\sum_{1}^{i} A_{i}\tau_{i}}$ 2

The absolute PL quantum yields (QYs) were recorded on a Quantaurus-QY Plus C13534-12 (Hamamatsu, Japan). The QY values were obtained according to the equation 3:

QY = $\frac{The number of photons emitted from the sample}{The number of photons absorbed by the sample} \times100\%$ 3

The radiative and non-radiative transition rates (k_r_ and k_nr_) were calculated using the equation 4 and 5:

k_r_ + k_nr_ = $\frac{1}{\tau}$ 4

QY = $\frac{k_{r}}{k_{r} + k_{nr}}$ 5

The transmission electron microscopy (TEM) images were obtained using a JEOL JEM-3200FS microscope. The elemental maps of Cd, Zn, Se and S were recorded on an energy disperse spectrometer (100TLE, X-MaxN). The powder X-ray diffraction (XRD) patterns were attained from an X-ray diffractometer (MiniFlex600, Rigaku Corporation). A surface analysis system (Nexsa G2, Thermo Scientific) was used to obtain the UPS (ultraviolet photoelectron spectroscopy) and XPS (X-ray photoelectron spectroscopy) spectra of the film samples. The electroluminescent (EL) spectra, luminances (*L*), current densities (*J*), current and power efficiencies (CEs/PEs), and external quantum efficiencies (EQEs) of devices were measured using an external quantum efficiency measurement system (C9920−12, Hamamatsu), which consists of a source meter (Keithley 2400), an integrating sphere, and a multi-channel analyzer PMA−12. A multi-channel OLED/QLED lifetime test system (D3000, Guanzhou Crysco Equipment Co., Ltd) was used to obtain the opertion lifetimes of devices and an electrochemical impedance spectrometer (SP-240, Bio-Logic SAS) was used to attain the Nyquist plots of devices.

1. **Supplementary Figures and Tables**

**a b c d**


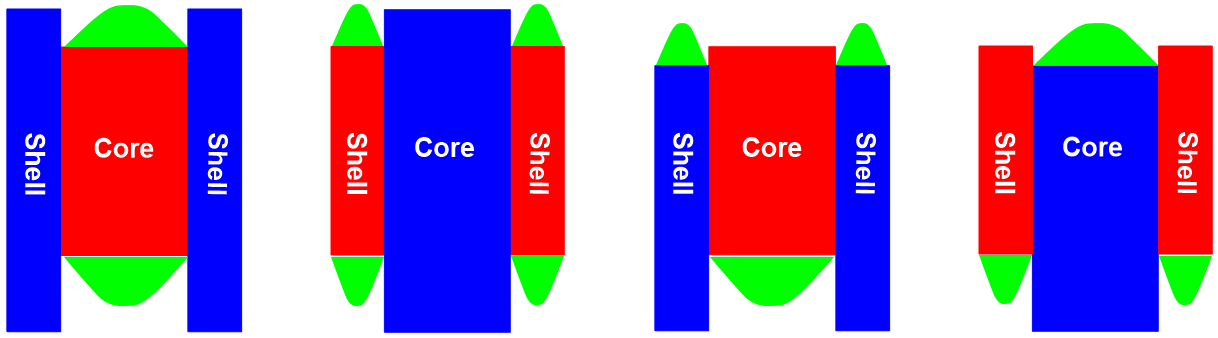


**e f**


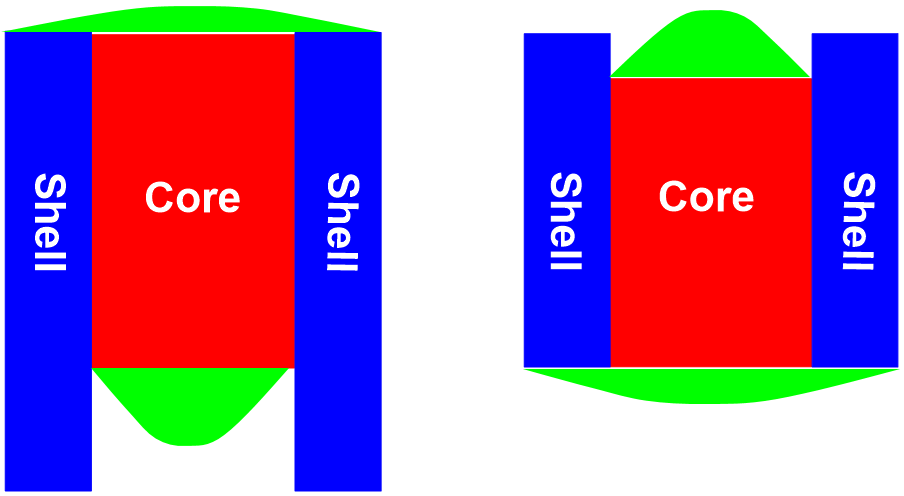


**Figure S1**. a and b, Conventional and reverse type-I energy level alignment, respectively. c and d, Type-II energy level alignment. e and f, Quasi-type-II energy level alignment.

In the type-I QDs, the wave functions of charge carriers are entirely confined inside the semiconductors with narrow band gaps, such as the cores in the conventional type-I QDs (Figure S1a) and the shells in the reverse type-I QDs (Figure S1b).^[5]^ The conventional type-I QDs can easily achieve near 100% PL QYs and narrow emission spectra.^[6]^ However, it is noted that the exposed emission centers are susceptible to the surface defects, resulting in the excited states of the reverse type-I QDs being easily quenched.^[5]^ In contrast to the type-I QDs, the wave functions of charge carriers are delocalized in the type-II and quasi-type-II structures.^[5]^ For the type-II structures, the charge carriers are separated in the cores or the shells. On the other hand, the charge carriers will be diffused in the entire core/shell in the quasi-type-II QDs. A recombination at the interface of core/shell occurs, affording the emission spectra of type-II QDs with multiple peaks originating from different semiconductors.^[7]^


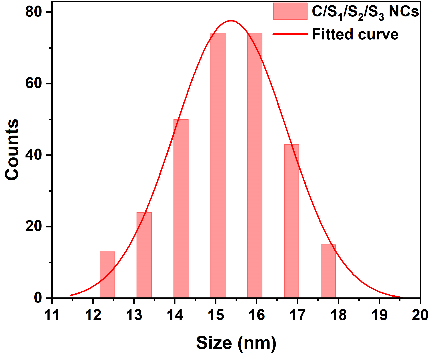


**Figure S2**. Size distribution of C/S_1_/S_2_/S_3_ NCs (mean size = 15.28 nm).

**a b**


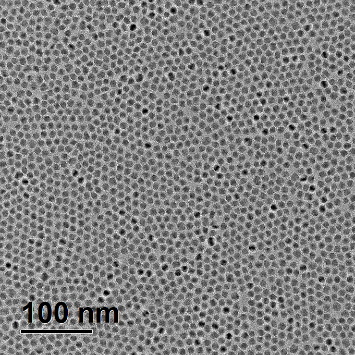

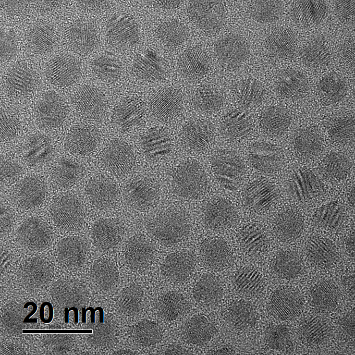


**c d**


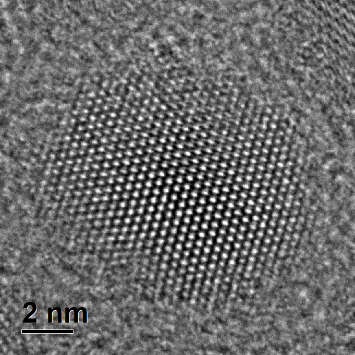

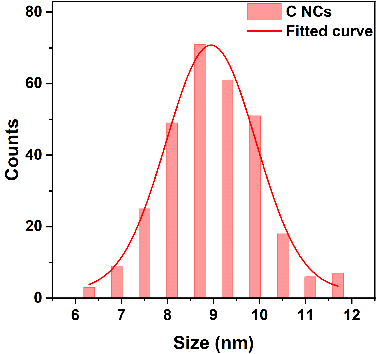


**Figure S3**. a−c, TEM images of C NCs with different scale bars. d, Size distribution of C NCs (mean size = 8.98 nm).

**a b**


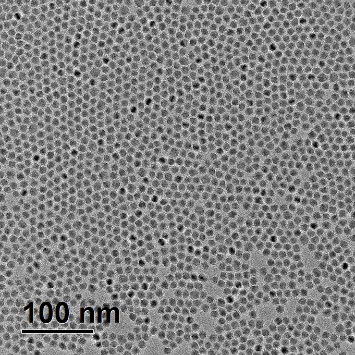

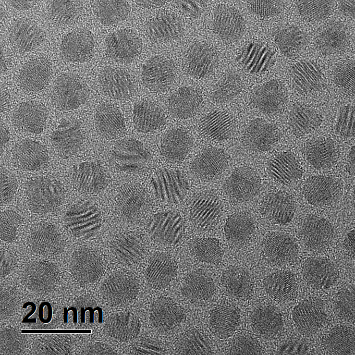


**c d**


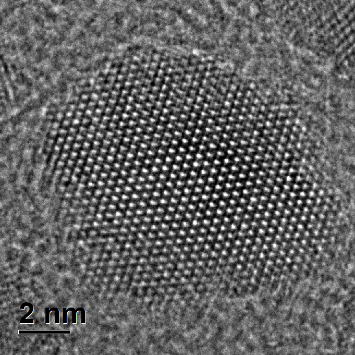

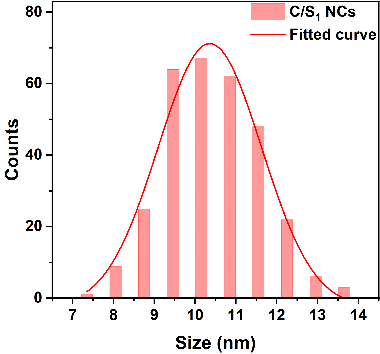


**Figure S4**. a−c, TEM images of C/S_1_ NCs with different scale bars. d, Size distribution of C/S_1_ NCs (mean size = 10.40 nm).

**a b**


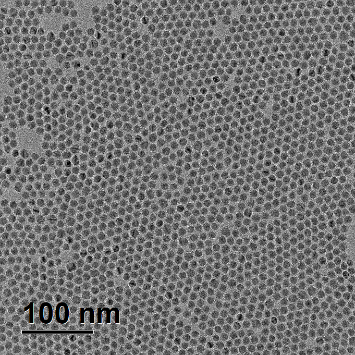

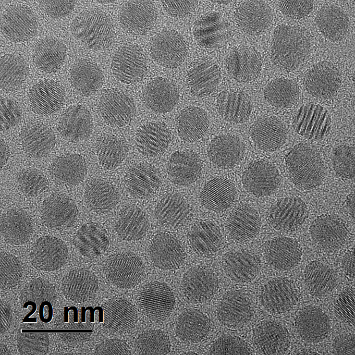


**c d**


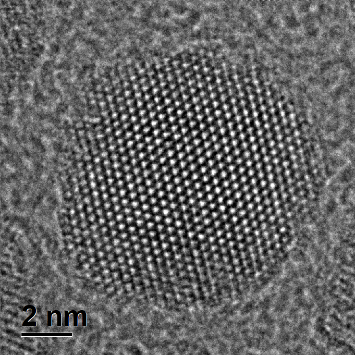

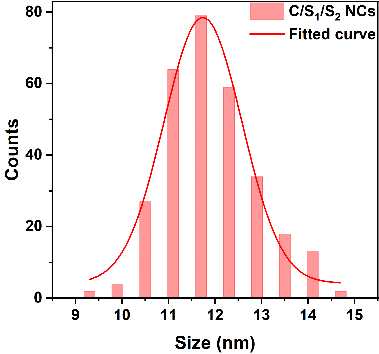


**Figure S5**. a−c, TEM images of C/S_1_/S_2_ NCs with different scale bars. d, Size distribution of C/S_1_/S_2_ NCs (mean size = 11.89 nm).


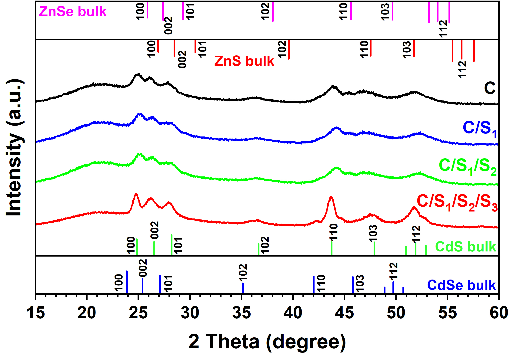


**Figure S6**. XRD patterns of NCs.

**Figure S7**. a−d, EDS elemental mapping of the C, C/S_1_, C/S_1_/S_2_ and C/S_1_/S_2_/S_3_ NCs, respectively. The red circles in a and b represent the region of CdSe distribution with a corresponding size of aroud 6.60 nm.

**Figure S8**. a−d, EDS reports of the C, C/S_1_, C/S_1_/S_2_ and C/S_1_/S_2_/S_3_ NCs, respectively.

**Figure S9**. XPS spectra of NCs. a, XPS survey. b and c, HR-XPS spectra of Cd 3d and Zn 2p, respectively. Notably, the Cd 3d signal strengthens as Zn 2p declines (Figure S9b and S9c), confirming the shift in composition from the inner Zn-rich domains (C and S_1_) to the outer Cd-rich areas (S_2_ and S_3_), in line with EDS results.

**Figure S10**. PL spectrum of the C/S_1_/S_2_/S_3_ QS: a, Spectral symmetry; and b, stability under UV radiation.

**Figure S11**. a−c, PL spectra, PL decay curves, and absolute PL QYs of the NC films, respectively.

**a b**


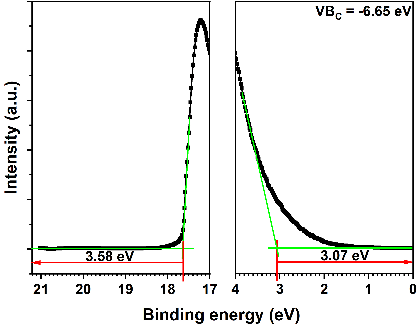

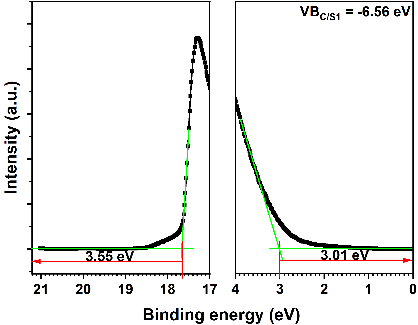


**c d**


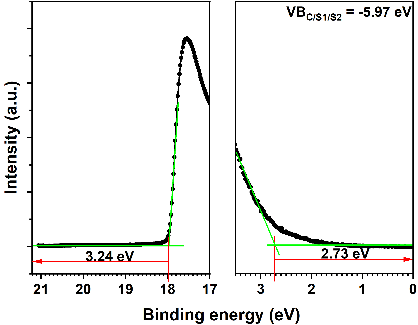

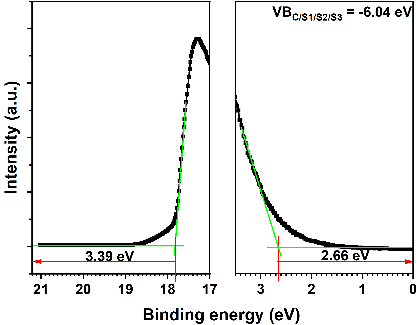


**Figure S12**. a−d, UPS spectra of the C, C/S_1_, C/S_1_/S_2_ and C/S_1_/S_2_/S_3_ NCs, respectively.

**a b c d**


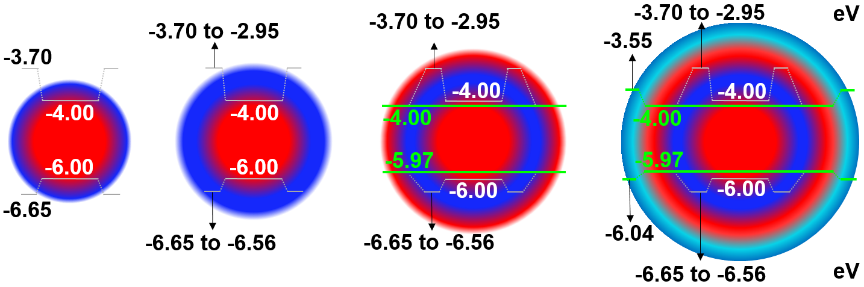


**Figure S13**. a−d, Schematic diagrams of energy level alignment for the C, C/S_1_, C/S_1_/S_2_ and C/S_1_/S_2_/S_3_ NCs, respectively. The VB energy level of the inner CdSe is about −6.00 eV according to the previous reports.^[8]^ To ensure clear distinction between energy levels, we have strategically separated these similar states in the diagram.

**Figure S14**. a and b, EL spectra of QS-LED measured at different applied voltages. c, Overlap of the EL and PL spectra.

**Figure S15**. Device performance of the QD- and QS-LEDs. a, EL spectra. b, *J*−*V*−*L* curves. c, CE and PE *vs* *L* curves. d, EQE *vs* *L* curves.


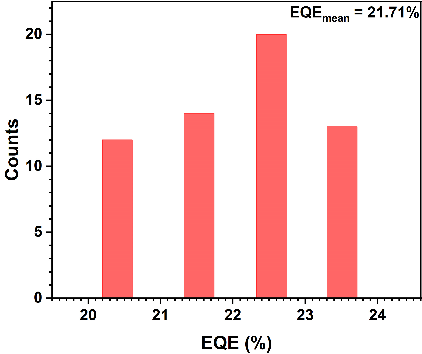


**Figure S16**. EQE reproducibility of QS-LEDs.

**Figure S17**. a and b, Operation stability of QD- and QS-LEDs, respectively. The inset represents the surface temperature (^o^C) of device measured at the initial luminance.

**Figure S18**. a and b, Charge carrier dynamics in the hole- and electron-only devices (HODs and EODs) based on the CdZnSe/ZnSeS/CdSeS/CdS QS, respectively. c and d, Charge carrier dynamics in the HOD and EOD based on the CdZnSe/ZnSeS/CdZnS QD, respectively. e, *J−V* curves of these HODs and EODs.

**Figure S19**. a and b, R_tr_ and R_rec_ in QD- and QS-LEDs, respectively. c, The experimental (symbols) and fitted (lines) EIS Nyquist plots of QD- and QS-LEDs. The inset in Figure S17c is the equivalent circuit used to fit these experimental data.

**Figure S20**. a and b, Square-wave signals used to control the time for the injection, transport and recombination of charge carriers in the light-emitting devices. Under a forward voltage (>*V*_on_), the LEDs will be lighted-up if the time is long enough to achieve the injection, transport and recombination of charge carriers. However, the LEDs fail to work under a reverse voltage. By setting a duty ratio of 50%, the time (t) for the charge carrier injection, transport and recombination is equal to the half of the period (T). Therefore, the t value could be calculated according to the equation of t = $\frac{\text{500}}{\text{f}}$ (ns), where f represents the frequency.

**a b**


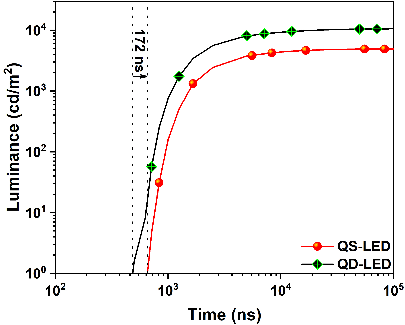

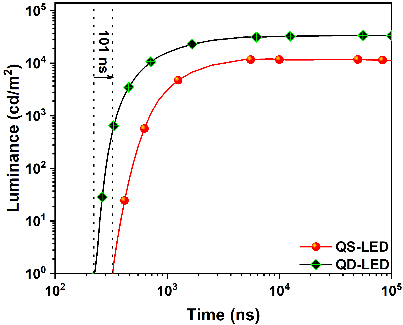


**Figure S21**. a and b, *L*−t curves of devices measured at 3 and 5 V.

**a**


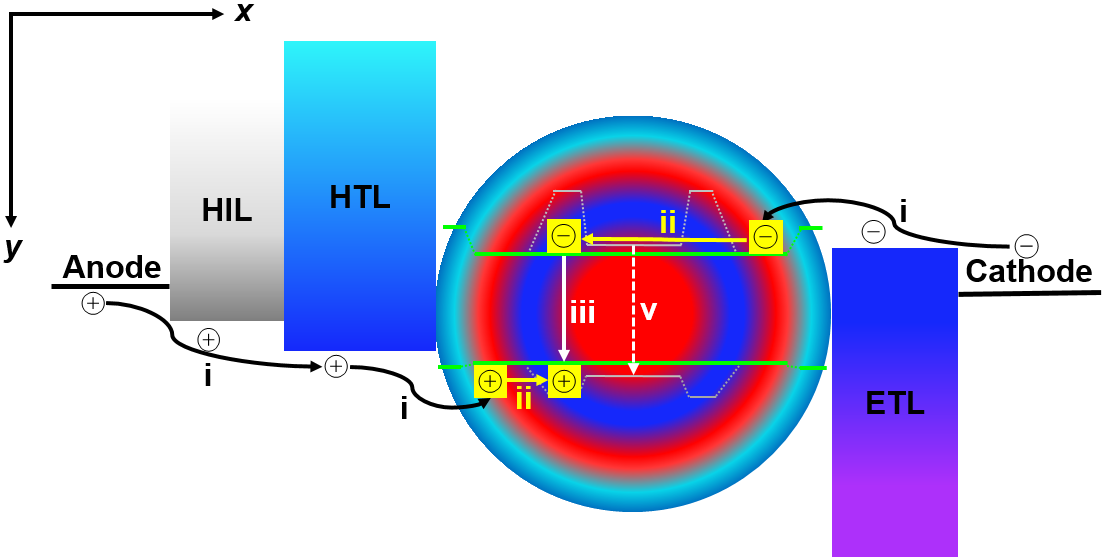


**b**


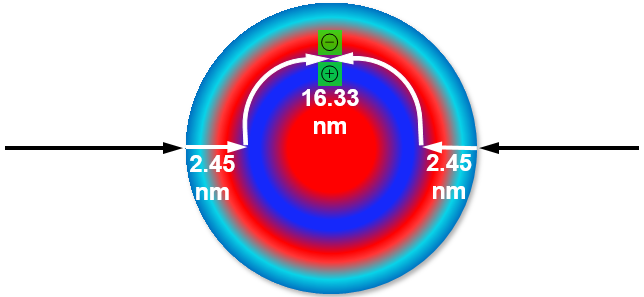


**c**


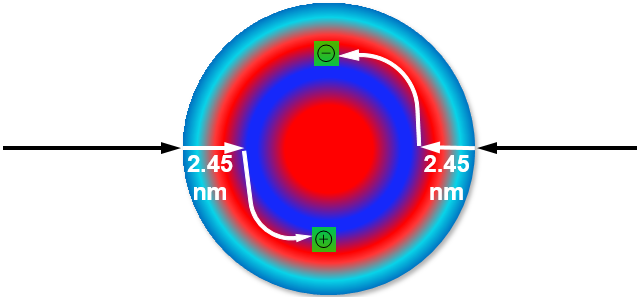


**Figure S22**. a, Schematic diagram for the dynamics in the QS-LED. b, One of the shortest paths taken by the electrons and holes from reaching the QS surface to the recombination site. c, One of the possible paths that the electrons and holes are injected inside QS but they fail to recombine immediately. Figure S20b and S20c depict the *y*-direction cross profiles of the energy level structure in Figure S20a.

**Figure S23**. a, Schematic diagram for the dynamics in the QD-LED. b, One of the shortest paths taken by the electrons and holes from reaching the QD surface to the recombination site. Figure S21b depicts the *y*-direction cross profiles of the energy level structure in Figure 21a.

**Table S1**. wt% of Cd, Zn, Se and S reported from the EDS spectra of NCs.

| NC | Cd [wt%] | Zn [wt%] | Se [wt%] | S [wt%] |
| --- | --- | --- | --- | --- |
| C | 25.1 | 30.1 | 25.1 | − |
| C/S_1_ | 21.5 | 33.8 | 42.8 | 1.8 |
| C/S_1_/S_2_ | 45.7 | 16.2 | 35.3 | 2.8 |
| C/S_1_/S_2_/S_3_ | 63.1 | 8.8 | 17.8 | 10.3 |

**Table S2**. PL properties of these NCs in this work.

| NC | λ_em_  [nm] | FWHM  [nm] | τ_av_  [ns] | QY  [%] | k_r_  [×10^6^ s^−1^] | k_nr_  [×10^6^ s^−1^] | *E*_g_  [eV] |
| --- | --- | --- | --- | --- | --- | --- | --- |
| C | 622^[a]^, 621^[b]^ | 23^[a]^, 24^[b]^ | 22.1^[a]^, 1.6^[b]^ | 25.4^[a]^, 5.0^[b]^ | 11.5^[a]^, 31.4^[b]^ | 33.7^[a]^, 597.5^[b]^ | 1.99 |
| C/S_1_ | 620^[a]^, 620^[b]^ | 22^[a]^, 24^[b]^ | 26.2^[a]^, 7.1^[b]^ | 78.2^[a]^, 46.7^[b]^ | 29.8^[a]^, 66.2^[b]^ | 8.3^[a]^, 75.5^[b]^ | 1.99 |
| C/S_1_/S_2_ | 629^[a]^, 627^[b]^ | 21^[a]^, 23^[b]^ | 137.6^[a]^, 43.3^[b]^ | 58.3^[a]^, 32.9^[b]^ | 4.2^[a]^, 7.6^[b]^ | 3.0^[a]^, 15.5^[b]^ | 1.97 |
| C/S_1_/S_2_/S_3_ | 630^[a]^, 629^[b]^ | 21^[a]^, 22^[b]^ | 215.2^[a]^, 135.2^[b]^ | 90.9^[a]^, 80.6^[b]^ | 4.2^[a]^, 6.0^[b]^ | 0.42^[a]^, 1.4^[b]^ | 1.97 |

[a] and [b] Measured in solutions and films, respectively.

**Table S3**. EL performance of the QD- and QS-LED.

| LED | *V*_on_^[a]^  [V] | *L* at 6 V  [×10^4^ cd m^−2^] | CE  [cd A^−1^] | PE  [lm W^−1^] | EQE  [%] | *T*_50_  [h] |
| --- | --- | --- | --- | --- | --- | --- |
| QD-LED | 1.7 | 10.81 | 31.14 | 45.29 | 21.78 | 81.2^[b]^ |
| QS-LED | 1.7 | 4.54 | 26.98 | 32.97 | 22.16 | 37.3^[c]^ |

[a] Corresponding to the applied voltage when the brightness of light-emitting device is 1 cd m^−2^. [b] and [c] The initial brightness values for the QD- and QS-LEDs are around 51240 and 33200 cd m^−2^, respectively.

**Table S4**. Fitting parameters of the EIS Nyquist plots.

| LED | R_s_  [Ω m^−2^] | R_tr_  [Ω m^−2^] | CPE_1_  [10^-9^ S Sec^n^ cm^−2^] | R_rec_  [Ω m^−2^] | CPE_2_  [10^-9^ S Sec^n^ cm^−2^] |
| --- | --- | --- | --- | --- | --- |
| QD-LED | 135 | 42 | 13.0 | 74119 | 2.3 |
| QS-LED | 125 | 146 | 14.9 | 128116 | 2.3 |

**References**

[1] B. Liu, Y. Guo, Q. Su, Y. Zhan, Z. Chen, Y. Li, B. You, X. Dong, S. Chen, W.-Y. Wong, *Adv. Sci.* **2022**, *9*, 2104488.

[2] X. Fan, Z. Mu, Z. Chen, Y. Zhan, F. Meng, Y. Li, G. Xing, W.-Y. Wong, *Chem. Eng. J.* **2023**, *461*, 142027.

[3] Z. Chen, X. Chen, Y. Zhang, Y. Zhan, G. Yuan, X. Lu, W. Zhang, Y. Li, X. Liu, F. Meng, *Ceram. Int.* **2024**, *50*, 28403.

[4] Z. Chen, N. Tian, X. Chen, Y. Zhang, B. Liu, Z. Yuan, C.-Y. He, A.-J, Wong, Y. Li, S. Ren, Z. Sun, W.-Y. Wong, *Adv. Mater.* **2025**, *37*, 2413978.

[5] P. Reiss, M. Protière, L. Li, *Small* **2009**, *5*, 154.

[6] Y. Gao, B. Li, X. Liu, H. Shen, Y. Song, J. Song, Z. Yan, X. Yan, Y. Chong, R. Yao, S. Wang, L. S. Li, F. Fan, Z. Du, *Nat.* *Nanotechnol.* **2023**, *18*, 1168.

[7] W. K. Bae, L. A. Padilha, Y.-S. Park, H. McDaniel, I. Robel, J. M. Pietryga, V. I. Klimov, *ACS Nano* **2013**, *7*, 3411.

[8] K. Tvrdy, P. A. Frantsuzov, P. V. Kamat, *P. Natl. Acad. Sci. UAS* **2011**, *108*, 29.
